# Ethyl 2-[*N*-(*tert*-butyl­sulfin­yl)carbamo­yl]benzoate

**DOI:** 10.1107/S1600536811047027

**Published:** 2011-11-19

**Authors:** Aurelien Honraedt, Sonia Ladeira, Thierry Berranger, Emmanuel Gras

**Affiliations:** a CNRS, LCC, 205 route de Narbonne, F-31077 Toulouse, France; b Université de Toulouse, UPS, INPT, LCC, F31077 Toulouse, France; c Minakem, 145 Chemin des Lilas, F-59310 Beuvry-La-Foret, France

## Abstract

The title compound, C_14_H_19_NO_4_S, was obtained in quanti­tative yield by Lewis acid-catalysed alcoholysis of a phtalimide precursor. An intra­molecular C—H⋯O hydrogen bond occurs. In the crystal, centrosymmetric dimers are formed by pairs of N—H⋯O hydrogen bonds between the sulfinyl O atoms and the carbamoyl N—H group of a neighboring mol­ecule. C—H⋯O inter­actions feature in the crystal structure.

## Related literature

For a related compound, see: Harpp & Back (1973 ▶). For hydrogen-bond motifs and graph-set notation, see: Etter (1990 ▶); Bernstein *et al.* (1995 ▶). For potential applications of the title compound in the synthesis of enones, see: Wang *et al.* (2005 ▶). For standard bond lengths, see: Allen *et al.* (1987 ▶). 
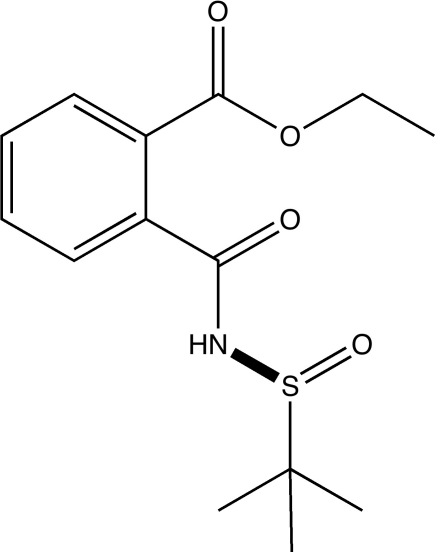


## Experimental

### 

#### Crystal data

C_14_H_19_NO_4_S
*M* *_r_* = 297.37
Monoclinic, 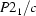

*a* = 11.7881 (3) Å
*b* = 9.0056 (2) Å
*c* = 16.3296 (4) Å
β = 120.091 (2)°
*V* = 1499.91 (7) Å^3^
*Z* = 4
Mo *K*α radiation
μ = 0.23 mm^−1^
*T* = 180 K
0.4 × 0.25 × 0.03 mm

#### Data collection

Oxford-Diffraction Gemini diffractometer
Absorption correction: multi-scan (*CrysAlis PRO*; Oxford Diffraction, 2010 ▶) *T*_min_ = 0.930, *T*_max_ = 0.990
15187 measured reflections
2744 independent reflections
2334 reflections with *I* > 2s(*I*)
*R*_int_ = 0.022

#### Refinement

*R*[*F* ^2^ > 2σ(*F* ^2^)] = 0.029
*wR*(*F* ^2^) = 0.088
*S* = 1.12
2744 reflections
188 parameters
1 restraint
H atoms treated by a mixture of independent and constrained refinement
Δρ_max_ = 0.50 e Å^−3^
Δρ_min_ = −0.28 e Å^−3^

### 

Data collection: *CrysAlis PRO* (Oxford Diffraction, 2010 ▶); cell refinement: *CrysAlis PRO*; data reduction: *CrysAlis PRO*; program(s) used to solve structure: *SIR92* (Altomare *et al.*, 1994 ▶); program(s) used to refine structure: *SHELXL97* (Sheldrick, 2008 ▶); molecular graphics: *Mercury* (Macrae *et al.*, 2008 ▶); software used to prepare material for publication: *WinGX* (Farrugia, 1999 ▶).

## Supplementary Material

Crystal structure: contains datablock(s) global, I. DOI: 10.1107/S1600536811047027/im2332sup1.cif

Structure factors: contains datablock(s) I. DOI: 10.1107/S1600536811047027/im2332Isup2.hkl

Supplementary material file. DOI: 10.1107/S1600536811047027/im2332Isup3.cml

Additional supplementary materials:  crystallographic information; 3D view; checkCIF report

## Figures and Tables

**Table 1:** Hydrogen-bond geometry (Å, °)

*D*—H⋯*A*	*D*—H	H⋯*A*	*D*⋯*A*	*D*—H⋯*A*
N1—H101⋯O4^i^	0.86 (1)	2.00 (1)	2.857 (2)	173 (2)
C5—H5⋯O2^ii^	0.95	2.59	3.469 (3)	155
C13—H13*C*⋯O2	0.98	2.37	3.345 (2)	174

Symmetry codes: (i) 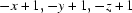; (ii) 
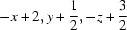
.

**Fig. 1.**
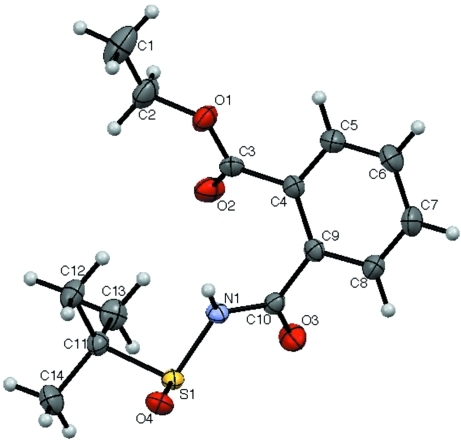
ORTEP representation of (I) (Mercury; Macrae et al., 2008) with ellipsoids drawn at the 30% probability level.

**Fig. 2.**
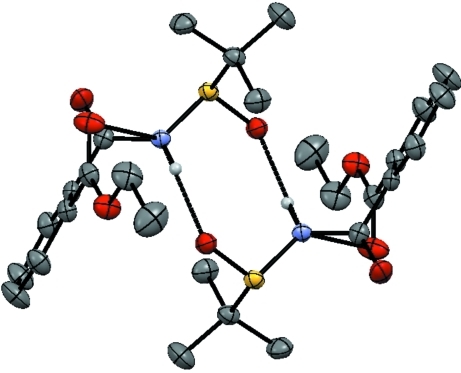
Dimeric structure showing the pair of intermolecular H–bonds and the chair like conformation of the core of the dimer with the two tert-butyl groups in axial positions. Only hydrogens involved in the H–bonds are represented; others were omitted for clarity.

## supplementary crystallographic information

### Comment

In the course of our studies on *tert*-butylsulfinyl phtalimide we have uncovered an unusual access to ethyl 2-(*tert*-butylsulfinylcarbamoyl)benzoate (I) based on the Lewis acid activation (for example by Samarium (III) salts) of one C=O bond of the phtalimide moiety. The title compound has been obtained quantitative yield.

Interestingly (I) exhibits one hydrogen donor (NH) and 5 electronegative atoms featuring available lone pairs that therefore should be able to act as hydrogen acceptors (4 oxygen and one sulfur). The combination of these donors and acceptors can induce a wide variety of hydrogen-bond patterns. A preferred one can be indicative of higher H-bond acceptor ability of one group.

Moreover, these features are of interest for potential applications in organocatalysis. Indeed acidic imide hydrogen atoms have been shown to favour organocatalytic processes in the formation of enones under mild reaction conditions. (Wang *et al.* 2005)

The crystal structure clearly establishes N–H···O contacts between the O atoms of the sulfinyl groups and the NH groups since the N···O distance falls by more than 0.2Å below he sum of the van-der-Waals radii of the atoms involved. This is strongly indicative of the presence of two intermolecular hydrogen bonds. From that point, a cyclic dimer is observed with a *R*_2_^2^(8) graph set (Fig. 2). Interestingly although isographic to the sulfinyl group (*i.e.* it has the same graph set but is chemically different), the carbonyl group of the amide is not involved in any hydrogen bond. This is a clear illustration of the higher polar character of the S=O bond making the sulfinyl oxygen a better H-bond acceptor than its carbonyl counterpart.

The *R*_2_^2^(8) graph set is a six bonds ring system exhibiting a chair like conformation in which the two *tert*-butyl groups are in axial positions and the two carbamoyl units in equatorial positions (Fig. 2). An unexpectedly small distance (in the range of the sum of the van-der-Waals radii) is observed between the oxygen of the carboxyl group of the ester and the nitrogen. The observed conformation might be minimizing the repulsive coulombic interaction and steric repulsions. All bond lengths and angles are otherwise normal. (Allen *et al.* 1987) Finally as the reaction has been carried out on racemic *tert*-butylsulfinyl phtalimide, (I) has been obtained as a racemate. It can be seen from the crystal structure that (I) crystallizes as a racemic compound (*i.e.* the two enantiomers forming dimers in the crystal lattice) indicating that no spontaneous resolution happens (formation of conglomerates).

### Experimental

To a suspension of 100 mg (0.398 mmol) of *tert*-butylsulfinyl phtalimide in 5 ml of ethanol stirred at room temperature is added a solution of 24 mg (0.04 mmol) of samarium(III) trifluoromethanesulfonate in 5 ml of ethanol. After 0.5 h of stirring at room temperature a complete solubilization is observed and a full conversion is confirmed by TLC (cyclohexane/diethyl ether: 2/8). After concentration of the reaction mixture under reduced pressure, the remainings are diluted with 10 ml of dichloromethane and washed twice with water (2x5 ml). The aqueous phases are extracted twice with 10 ml of dichloromethane. The combined organic phases are dried of sodium sulfate and concentrated to dryness under reduced pressure to give 118.1 mg (100% yield) of a white solid. Crystals of (I) suitable for X-ray diffraction were grown overnight at -20 °C in a 95/5 Diethyl ether/dichloromethane mixture.

### Refinement

All H atoms attached to C atoms were fixed geometrically and treated as riding with C—H = 0.98 Å (methyl) or 0.95 Å (aromatic) or 0.97 Å (methylene) with *U*_iso_(H) = 1.2*U*_eq_(C). The H attached to nitrogen has been located on difference Fourier and its coordinates were refined using N—H restraints of 0.88 (1) Å with *U*_iso_(H) = 1.2*U*_eq_(N).

### Crystal data

**Table:**

C_14_H_19_NO_4_S	*F*(000) = 632
*M**_r_* = 297.37	*D*_x_ = 1.317 Mg m^−^^3^
Monoclinic, *P*2_1_/*c*	Mo *K*α radiation, λ = 0.71073 Å
Hall symbol: -P 2ybc	Cell parameters from 9878 reflections
*a* = 11.7881 (3) Å	θ = 2.9–29.0°
*b* = 9.0056 (2) Å	µ = 0.23 mm^−^^1^
*c* = 16.3296 (4) Å	*T* = 180 K
β = 120.091 (2)°	Block, colourless
*V* = 1499.91 (7) Å^3^	0.4 × 0.25 × 0.03 mm
*Z* = 4	

### Data collection

**Table:**

Oxford-Diffraction Gemini diffractometer	2744 independent reflections
Radiation source: Enhance (Mo) X-ray Source	2334 reflections with *I* > 2s(*I*)
graphite	*R*_int_ = 0.022
ω scan	θ_max_ = 25.4°, θ_min_ = 2.9°
Absorption correction: multi-scan (*CrysAlis PRO*; Oxford Diffraction, 2010)	*h* = −14→14
*T*_min_ = 0.930, *T*_max_ = 0.990	*k* = −10→10
15187 measured reflections	*l* = −19→18

### Refinement

**Table:**

Refinement on *F*^2^	Primary atom site location: structure-invariant direct methods
Least-squares matrix: full	Secondary atom site location: difference Fourier map
*R*[*F*^2^ > 2σ(*F*^2^)] = 0.029	Hydrogen site location: inferred from neighbouring sites
*wR*(*F*^2^) = 0.088	H atoms treated by a mixture of independent and constrained refinement
*S* = 1.12	*w* = 1/[σ^2^(*F*_o_^2^) + (0.0508*P*)^2^ + 0.2576*P*] where *P* = (*F*_o_^2^ + 2*F*_c_^2^)/3
2744 reflections	(Δ/σ)_max_ < 0.001
188 parameters	Δρ_max_ = 0.50 e Å^−^^3^
1 restraint	Δρ_min_ = −0.28 e Å^−^^3^

### Special details

**Table:**

Experimental. 1H NMR (CDCl3, 400?MHz): 8.01 (s, 1H); 7.61–7.55 (m, 4H); 4.40 (q, J 7.2?Hz, 2H); 1.40 (t, J 7.2?Hz, 3H); 1.33 (s, 9H). 13C NMR (CDCl3, 100.6?MHz, T= 233?K): 171.8; 165.6; 136.1; 132.5; 130.5; 130.1; 129.1; 127.6; 62.0; 57.8; 22.4; 14.0. MS (DCI, NH3): 315.0; IR (cm-1): 3068; 2971; 1710; 1688; 1428; 1247; 1062; 885;805; 703. MP: 129–131 °C.
Geometry. All e.s.d.'s (except the e.s.d. in the dihedral angle between two l.s. planes) are estimated using the full covariance matrix. The cell e.s.d.'s are taken into account individually in the estimation of e.s.d.'s in distances, angles and torsion angles; correlations between e.s.d.'s in cell parameters are only used when they are defined by crystal symmetry. An approximate (isotropic) treatment of cell e.s.d.'s is used for estimating e.s.d.'s involving l.s. planes.
Refinement. Refinement of *F*^2^ against ALL reflections. The weighted *R*-factor *wR* and goodness of fit *S* are based on *F*^2^, conventional *R*-factors *R* are based on *F*, with *F* set to zero for negative *F*^2^. The threshold expression of *F*^2^ > σ(*F*^2^) is used only for calculating *R*-factors(gt) *etc*. and is not relevant to the choice of reflections for refinement. *R*-factors based on *F*^2^ are statistically about twice as large as those based on *F*, and *R*- factors based on ALL data will be even larger.

### Fractional atomic coordinates and isotropic or equivalent isotropic displacement parameters (Å^2^)

**Table:**

	*x*	*y*	*z*	*U*_iso_*/*U*_eq_	
C1	0.9715 (2)	0.8337 (3)	0.54534 (15)	0.0587 (6)	
H1A	0.8812	0.8463	0.4934	0.088*	
H1B	1.0281	0.8081	0.5195	0.088*	
H1C	1.0021	0.9265	0.5813	0.088*	
C2	0.97629 (19)	0.7127 (2)	0.60890 (14)	0.0419 (4)	
H2A	0.9451	0.6184	0.5733	0.05*	
H2B	1.0673	0.6982	0.6614	0.05*	
C3	0.87361 (14)	0.65483 (17)	0.69751 (10)	0.0262 (3)	
C4	0.79014 (14)	0.71356 (17)	0.73504 (10)	0.0241 (3)	
C5	0.79902 (17)	0.86253 (18)	0.76008 (12)	0.0328 (4)	
H5	0.8554	0.9269	0.7507	0.039*	
C6	0.72634 (19)	0.9176 (2)	0.79856 (13)	0.0409 (4)	
H6	0.7317	1.0197	0.8146	0.049*	
C7	0.64604 (18)	0.8236 (2)	0.81356 (12)	0.0386 (4)	
H7	0.5965	0.8611	0.8403	0.046*	
C8	0.63737 (16)	0.67464 (19)	0.78977 (11)	0.0296 (4)	
H8	0.5822	0.6106	0.8006	0.036*	
C9	0.70872 (14)	0.61845 (16)	0.75018 (10)	0.0224 (3)	
C10	0.68801 (14)	0.45713 (16)	0.72159 (10)	0.0224 (3)	
C11	0.68034 (15)	0.22686 (16)	0.52516 (10)	0.0255 (3)	
C12	0.67874 (18)	0.35133 (19)	0.46180 (12)	0.0344 (4)	
H12A	0.7299	0.3216	0.4322	0.052*	
H12B	0.7172	0.441	0.4998	0.052*	
H12C	0.5881	0.3717	0.4125	0.052*	
C13	0.81679 (16)	0.20074 (19)	0.60975 (12)	0.0350 (4)	
H13A	0.8755	0.1665	0.5875	0.052*	
H13B	0.8121	0.1253	0.6512	0.052*	
H13C	0.8505	0.2937	0.645	0.052*	
C14	0.62431 (19)	0.08368 (19)	0.46900 (13)	0.0410 (4)	
H14A	0.5337	0.1007	0.4186	0.062*	
H14B	0.6261	0.0055	0.5114	0.062*	
H14C	0.6772	0.053	0.441	0.062*	
N1	0.63756 (12)	0.43515 (13)	0.62653 (8)	0.0215 (3)	
H101	0.6177 (16)	0.5099 (14)	0.5890 (10)	0.026*	
O1	0.89198 (11)	0.75638 (12)	0.64578 (8)	0.0332 (3)	
O2	0.92023 (11)	0.53207 (12)	0.71343 (9)	0.0365 (3)	
O3	0.70681 (12)	0.35692 (12)	0.77681 (7)	0.0352 (3)	
O4	0.44238 (10)	0.30802 (11)	0.49210 (7)	0.0278 (3)	
S1	0.57360 (4)	0.27051 (4)	0.57391 (2)	0.02149 (13)	

### Atomic displacement parameters (Å^2^)

**Table:**

	*U*^11^	*U*^22^	*U*^33^	*U*^12^	*U*^13^	*U*^23^
C1	0.0548 (13)	0.0834 (16)	0.0556 (12)	0.0132 (12)	0.0408 (11)	0.0184 (12)
C2	0.0404 (10)	0.0503 (11)	0.0517 (11)	0.0016 (8)	0.0355 (9)	−0.0005 (9)
C3	0.0211 (8)	0.0294 (9)	0.0285 (8)	−0.0069 (6)	0.0128 (6)	−0.0054 (6)
C4	0.0244 (8)	0.0265 (8)	0.0219 (7)	−0.0029 (6)	0.0119 (6)	−0.0028 (6)
C5	0.0379 (9)	0.0297 (9)	0.0376 (9)	−0.0096 (7)	0.0239 (8)	−0.0078 (7)
C6	0.0533 (12)	0.0303 (9)	0.0514 (11)	−0.0087 (8)	0.0353 (10)	−0.0161 (8)
C7	0.0458 (11)	0.0393 (10)	0.0439 (10)	−0.0035 (8)	0.0325 (9)	−0.0124 (8)
C8	0.0317 (9)	0.0352 (9)	0.0277 (8)	−0.0055 (7)	0.0192 (7)	−0.0033 (7)
C9	0.0215 (7)	0.0265 (8)	0.0162 (7)	−0.0014 (6)	0.0073 (6)	−0.0015 (6)
C10	0.0196 (7)	0.0265 (8)	0.0226 (7)	−0.0011 (6)	0.0116 (6)	0.0008 (6)
C11	0.0301 (8)	0.0229 (8)	0.0280 (8)	0.0013 (6)	0.0180 (7)	−0.0009 (6)
C12	0.0413 (10)	0.0367 (10)	0.0358 (9)	0.0056 (7)	0.0272 (8)	0.0076 (7)
C13	0.0321 (9)	0.0341 (9)	0.0390 (9)	0.0102 (7)	0.0180 (8)	0.0038 (7)
C14	0.0496 (11)	0.0339 (9)	0.0465 (10)	−0.0044 (8)	0.0294 (9)	−0.0145 (8)
N1	0.0263 (7)	0.0169 (6)	0.0204 (6)	−0.0014 (5)	0.0111 (5)	0.0015 (5)
O1	0.0352 (7)	0.0354 (6)	0.0407 (6)	0.0007 (5)	0.0276 (6)	0.0012 (5)
O2	0.0307 (6)	0.0293 (6)	0.0570 (8)	0.0010 (5)	0.0275 (6)	−0.0006 (5)
O3	0.0486 (7)	0.0286 (6)	0.0267 (6)	−0.0010 (5)	0.0177 (5)	0.0059 (5)
O4	0.0244 (6)	0.0241 (5)	0.0290 (6)	−0.0026 (4)	0.0090 (5)	−0.0013 (4)
S1	0.0251 (2)	0.01767 (19)	0.0227 (2)	−0.00142 (13)	0.01277 (16)	0.00053 (13)

### Geometric parameters (Å, °)

**Table:**

C1—C2	1.486 (3)	C9—C10	1.508 (2)
C1—H1A	0.98	C10—O3	1.2150 (18)
C1—H1B	0.98	C10—N1	1.3699 (19)
C1—H1C	0.98	C11—C12	1.519 (2)
C2—O1	1.451 (2)	C11—C13	1.524 (2)
C2—H2A	0.99	C11—C14	1.527 (2)
C2—H2B	0.99	C11—S1	1.8373 (16)
C3—O2	1.2036 (19)	C12—H12A	0.98
C3—O1	1.3343 (19)	C12—H12B	0.98
C3—C4	1.493 (2)	C12—H12C	0.98
C4—C5	1.391 (2)	C13—H13A	0.98
C4—C9	1.398 (2)	C13—H13B	0.98
C5—C6	1.384 (2)	C13—H13C	0.98
C5—H5	0.95	C14—H14A	0.98
C6—C7	1.380 (3)	C14—H14B	0.98
C6—H6	0.95	C14—H14C	0.98
C7—C8	1.386 (2)	N1—S1	1.6898 (12)
C7—H7	0.95	N1—H101	0.860 (9)
C8—C9	1.388 (2)	O4—S1	1.4898 (11)
C8—H8	0.95		
			
C2—C1—H1A	109.5	O3—C10—C9	123.02 (13)
C2—C1—H1B	109.5	N1—C10—C9	113.63 (12)
H1A—C1—H1B	109.5	C12—C11—C13	112.22 (14)
C2—C1—H1C	109.5	C12—C11—C14	111.10 (13)
H1A—C1—H1C	109.5	C13—C11—C14	110.86 (14)
H1B—C1—H1C	109.5	C12—C11—S1	111.09 (11)
O1—C2—C1	107.28 (15)	C13—C11—S1	106.33 (10)
O1—C2—H2A	110.3	C14—C11—S1	104.89 (11)
C1—C2—H2A	110.3	C11—C12—H12A	109.5
O1—C2—H2B	110.3	C11—C12—H12B	109.5
C1—C2—H2B	110.3	H12A—C12—H12B	109.5
H2A—C2—H2B	108.5	C11—C12—H12C	109.5
O2—C3—O1	124.39 (15)	H12A—C12—H12C	109.5
O2—C3—C4	124.08 (14)	H12B—C12—H12C	109.5
O1—C3—C4	111.52 (13)	C11—C13—H13A	109.5
C5—C4—C9	119.75 (14)	C11—C13—H13B	109.5
C5—C4—C3	119.63 (14)	H13A—C13—H13B	109.5
C9—C4—C3	120.52 (13)	C11—C13—H13C	109.5
C6—C5—C4	120.43 (15)	H13A—C13—H13C	109.5
C6—C5—H5	119.8	H13B—C13—H13C	109.5
C4—C5—H5	119.8	C11—C14—H14A	109.5
C7—C6—C5	119.79 (16)	C11—C14—H14B	109.5
C7—C6—H6	120.1	H14A—C14—H14B	109.5
C5—C6—H6	120.1	C11—C14—H14C	109.5
C6—C7—C8	120.32 (16)	H14A—C14—H14C	109.5
C6—C7—H7	119.8	H14B—C14—H14C	109.5
C8—C7—H7	119.8	C10—N1—S1	122.17 (10)
C7—C8—C9	120.43 (15)	C10—N1—H101	120.2 (11)
C7—C8—H8	119.8	S1—N1—H101	115.6 (11)
C9—C8—H8	119.8	C3—O1—C2	116.06 (13)
C8—C9—C4	119.29 (14)	O4—S1—N1	104.78 (6)
C8—C9—C10	116.97 (14)	O4—S1—C11	106.78 (7)
C4—C9—C10	123.68 (13)	N1—S1—C11	100.38 (7)
O3—C10—N1	123.21 (14)		

### Hydrogen-bond geometry (Å, °)

**Table:**

*D*—H···*A*	*D*—H	H···*A*	*D*···*A*	*D*—H···*A*
N1—H101···O4^i^	0.86 (1)	2.00 (1)	2.857 (2)	173 (2)
C5—H5···O2^ii^	0.95	2.59	3.469 (3)	155.
C13—H13C···O2	0.98	2.37	3.345 (2)	174.

Symmetry codes: (i) −*x*+1, −*y*+1, −*z*+1; (ii) −*x*+2, *y*+1/2, −*z*+3/2.

## References

[bb1] Allen, F. H., Kennard, O., Watson, D. G., Brammer, L., Orpen, A. G. & Taylor, R. (1987). *J. Chem. Soc. Perkin Trans. 2*, pp. S1–19.

[bb2] Altomare, A., Cascarano, G., Giacovazzo, C., Guagliardi, A., Burla, M. C., Polidori, G. & Camalli, M. (1994). *J. Appl. Cryst.* **27**, 435.

[bb3] Bernstein, J., Davis, R. E., Shimoni, L. & Chang, N.-L. (1995). *Angew. Chem. Int. Ed. Engl.* **34**, 1555–1573.

[bb4] Etter, M. C. (1990). *Acc. Chem. Res.* **23**, 120–126.

[bb5] Farrugia, L. J. (1999). *J. Appl. Cryst.* **32**, 837–838.

[bb6] Harpp, D. N. & Back, T. G. (1973). *J. Org. Chem.* **38**, 4328–4334.

[bb7] Macrae, C. F., Bruno, I. J., Chisholm, J. A., Edgington, P. R., McCabe, P., Pidcock, E., Rodriguez-Monge, L., Taylor, R., van de Streek, J. & Wood, P. A. (2008). *J. Appl. Cryst.* **41**, 466–470.

[bb8] Oxford Diffraction (2010). *CrysAlis PRO* Oxford Diffraction Ltd, Yarnton, England.

[bb9] Sheldrick, G. M. (2008). *Acta Cryst.* A**64**, 112–122. 10.1107/S0108767307043930 18156677

[bb10] Wang, W., Mei, Y., Li, H. & Wang, J. (2005). *Org. Lett.* **7**, 601–604. 10.1021/ol047630g 15704904

